# Defining and conceptualising data harmonisation: a scoping review protocol

**DOI:** 10.1186/s13643-018-0890-7

**Published:** 2018-12-06

**Authors:** Bey-Marrié Schmidt, Christopher J. Colvin, Ameer Hohlfeld, Natalie Leon

**Affiliations:** 1 School of Public Health and Family Medicine, Faculty of Health Sciences, University of Cape Town, Falmouth Rd, Observatory, Cape Town, 7925 South Africa; 2 Cochrane South Africa, South African Medical Research Council, Cape Town, South Africa; 3 Division of Social and Behavioural Sciences, School of Public Health and Family Medicine, University of Cape Town, Cape Town, South Africa; 4 Department of Epidemiology, School of Public Health, Brown University, Providence, USA; 5 Department of Public Health Sciences, University of Virginia, Charlottesville, USA; 6 Health Systems Research Unit, South African Medical Research Council, Cape Town, South Africa

**Keywords:** Data harmonisation, Data linkage, Health information exchange, Routine health information system, Scoping review, Health management decision-making

## Abstract

**Background:**

Data harmonisation is an important intervention to strengthen health systems functioning. It has the potential to enhance the production, accessibility and utilisation of routine health information for clinical and service management decision-making. It is important to understand the range of definitions and concepts of data harmonisation, as well as how its various social and technical components and processes are thought to lead to better health management decision-making. However, there is lack of agreement in the literature, and in practice, on definitions and conceptualisations of data harmonisation, making it difficult for health system decision-makers and researchers to design, implement, evaluate and compare data harmonisation interventions. This scoping review aims to synthesise (1) definitions and conceptualisations of data harmonisation as well as (2) explanations in the literature of the causal relationships between data harmonisation and health management decision-making.

**Methods:**

This review follows recommended methodological stages for scoping studies. We will identify relevant studies (peer-reviewed and grey literature) from 2000 onwards, in English only, and with no methodological restriction, in various electronic databases, such as CINAHL, MEDLINE via PubMed and Global Health. Two reviewers will independently screen records for potential inclusion for the abstract and full-text screening stages. One reviewer will do the data extraction, analysis and synthesis, with built-in reliability checks from the rest of the team. We will use a combination of sampling techniques, including two types of ‘purposeful sampling’, a methodological approach that is particularly suitable for a scoping review with our objectives. We will provide (a) a numerical synthesis of characteristics of the included studies and (b) a narrative synthesis of definitions and explanations in the literature of the relationship between data harmonisation and health management decision-making.

**Discussion:**

We list potential limitations of this scoping review. To our knowledge, this scoping review will be the first to synthesise definitions and conceptualisations of data harmonisation in the literature as well as the underlying explanations in the literature of the causal links between data harmonisation and health management decision-making.

**Electronic supplementary material:**

The online version of this article (10.1186/s13643-018-0890-7) contains supplementary material, which is available to authorized users.

## Background

An effective health system relies on a routine health information system (RHIS) that provides the informational support needed by health managers to identify gaps in service delivery and to inform planning, implementation and monitoring of interventions [1]. However, many countries, especially in low-and middle-income settings, do not have well-functioning routine health information systems (RHISs) to monitor and evaluate their work [2, 3]. This limits countries’ ability to improve the effectiveness, efficiency, quality and equity of their health services.

Given the increasing availability of large electronic databases of routine health information, health authorities and managers, information technology (IT) stakeholders and researchers have identified data harmonisation as an important intervention for strengthening routine health information systems (RHISs) [4, 5]. There is often a lack of coordination and integration of large electronic databases; this is typically due to inconsistencies between key variables and indicators for collecting, analysing and reporting health information across programmes [8]; the production of poor quality data that cannot easily be exchanged [6]; and programmatic fragmentation across levels of the health system which can result in the duplication and excessive production of data [9]. Data harmonisation has the potential to address all these problems, through coordination, linkage and integration of existing large-scale databases [6–8].

Harmonised data sets also have the potential to improve informational support for health management decision-making and, in turn, support health systems strengthening [9, 10]. However, data harmonisation interventions may take on different parts of the problem of fragmented systems, use different definitions and may have different intended outcomes with regard to improving routine health information systems. This makes comparison and assessment of its usefulness for improving health systems functioning difficult to assess. A second challenge is that sometimes even when quality and timely health information are available, limited access to and use by management for planning, monitoring and evaluation and quality improvement is still a problem [6, 7, 9]. Data harmonisation has the potential to provide timely, relevant and accessible informational support for health management decision-making [12, 13], but we need to better understand how data harmonisation might actually work to improve decision-making. In this review, we are interested to learn more about the scope of data harmonisation definitions and activities as well as how those working in this field understand its effect on management decision-making. Our assumption is that harmonised routine health information may increase access to and use of relevant routine health information which could improve management decision-making and tasks of monitoring, evaluation, planning and ongoing quality improvement. We define effective management decision-making as the proactive and interactive process that demands and uses the best available data (well-integrated, complete and accurate data) during programme development as well as monitoring and evaluation [9].

### Why it is important to do this scoping review

There is growing recognition that the successful implementation of data harmonisation interventions occurs in multiple technical and social (i.e. organisational and behavioural) contexts. This multi-faceted nature of data harmonisation has resulted in a range of different terms being used for interventions with similar aims and activities [11]. For example, terms such as *data integration* [12], *data linkage* [13] and *health information exchange* [10] are all used to describe data harmonisation-type activities, and it is not always clear the extent to which these efforts are similar in practice, scope and relevance. While the use of multiple terms is not a problem in itself, lack of clarity on what constitutes ‘data harmonisation’ makes it difficult to compare studies and synthesise evidence on impact.

Lack of understanding of the underlying causal mechanisms between the data harmonisation activities and the intended outcomes for health management decision-making also makes it difficult to compare interventions and to evaluate the impact and implications for health systems strengthening. Having a clearer idea of the range of definitions and concepts used, the various components and activities included in data harmonisation interventions and the proposed underlying causal mechanisms being tested can help inform researchers and health system decision-makers on the design, implementation and evaluation of different data harmonisation interventions [14].

Since 2012, there have been three systematic reviews on data harmonisation and related activities, indicating a growing interest in the topic. The reviews were concerned with the integration of health information found in multiple databases across multiple organisations, for the purposes of clinical and service improvements, and for research analyses. One review focused on the determinants of RHIS performance and its role in improving health systems functioning and performance at the local level [9]. Another focused on views of health care professionals on data sharing or data linkage of clinical data for research purposes [8], while the third focused on barriers and facilitators of health information exchange (HIE) in LMICs [12]. Consistent with what was found in primary studies of data harmonisation processes, these reviews used a variety of terms to explain the integration and exchange of health information [15]. Data harmonisation was defined both narrowly and broadly depending on its objectives; in one review, *data linkage* was used solely to describe the technical stages of combining multiple databases [8], while in another, *health information exchange* was used to describe similar as well as broader processes involving multiple stakeholders to mobilise information across various systems, organisations and geographical areas [16]. It is important to identify and synthesise these variations in terminology in a systematic way, to reflect both the range of activities, but also to identify the commonalities, and build an understanding of how data harmonisation interventions are thought to work to support the different needs of implementers and/or users of harmonised data.

## Methods

This scoping review will follow the methodological stages for scoping studies proposed by Arksey and O’Malley [15] who recommend a process that is “not linear but iterative, requiring researchers to engage with each stage in a reflexive way” in order to achieve both ‘in-depth and broad’ results. The steps involved are identifying the research question, identifying relevant studies, selecting studies for inclusion, data extraction and data synthesis.

### Study question and objectives

This scoping review aims to appraise the characteristics of studies on data harmonisation and the definitions used for data harmonisation activities and to develop an understanding of the intended effect of data harmonisation interventions on management decision-making. The objectives are:

1. To identify and synthesise the characteristics of studies of data harmonisation;
2. To identify and synthesise the various definitions and concepts used to describe data harmonisation interventions, and
3. To develop a conceptual understanding of explanations in the literature of the causal relationship between data harmonisation interventions and health management decision-making.

In order to inform our understanding of the causal mechanisms (including the role of key contextual socio-technical dynamics) (objective 3), we will draw on information extracted for objectives 1 and 2 and, in addition, extract data on the descriptions of the components, processes, contexts and intended causal pathways of data harmonisation interventions. Such a synthesis has the potential to broaden and clarify the knowledge base of researchers and health management about the range of and variation in data harmonisation interventions, and the intended relationship between the components (individually or in combination) and management decision-making.

### Identifying relevant studies

#### Eligibility criteria

Peer-reviewed research studies (no methodological restrictions) and grey literature on data harmonisation in health-related information databases are eligible if they provide (a) a definition and/or a conceptualisation of data harmonisation (and/or related terms) and/or (b) a description of a data harmonisation intervention (in terms of components and processes and causal mechanisms) and/or (c) contribute to an explanation of the causal relationship between data harmonisation and health management decision-making (for example, through improved quality and accessibility of harmonised information for management and or the utilisation of harmonised health information for management decision-making). Studies concerned with various technical aspects of data harmonisation, such as changes in key variables and indicators, software and hardware infrastructure for data generation, and in reporting and feedback procedures, are also eligible, provided it is considered part of a data harmonisation intervention.

#### Search strategy

The search will identify all relevant studies from the year 2000 onwards (01 January 2000 to 31 July 2018). This is around the time that large-scale digitisation of routine information started to be implemented (especially in LMICs), and when policy-makers and researchers became interested in harmonisation of large digital databases [2, 3, 9, 16]. The following electronic databases will be searched for eligible studies:

- CINAHL, EbscoHOST
- MEDLINE via PubMed
- Global Health
- Science Citation Index and Social Sciences Citation Index, ISI Web of Science
- Relevant websites, such as the World Health Organization (WHO) and MEASURE Evaluation websites

Search terms will include a distillation of keywords and Medical Subject Headings (MeSH) terms related to data harmonisation (concept A) and health information system (concept B). We have developed a preliminary search strategy using relevant keywords and MeSH terms (see Additional file 1). To ensure that we do not miss potential studies, we will apply an iterative approach using known studies that meet the inclusion criteria identified during preparation of the protocol. Studies known to meet the inclusion criteria will be searched for among “hits” (search records) and used to identify new keywords and MeSH terms not already included in the search strategy. Once the search strategy has been finalised using the PubMed database, we will tailor it to each database and report on the adaptations. Searches will be limited to English as we do not have the resources required for reviewing non-English literature. There will be no geographic restrictions.

In addition to the electronic searches, review authors will (a) search the reference lists of all included studies and key references (for example, relevant systematic reviews) and (b) contact authors of included studies and/or experts in the field for additional references.

### Selecting studies for inclusion

#### Screening records

The initial search from different sources will be conducted to identify a database of records (title and abstracts) of relevant studies. The search results will be collated in the Endnote reference management programme and duplicates removed [17]. The final search database will then be uploaded into Covidence, an electronic programme designed for managing the screening process in systematic reviews (https://www.covidence.org). Two reviewers (BS and AH) will then independently screen the records to evaluate their eligibility for full-text review. The full texts of those studies identified as potentially relevant will be retrieved and read by the two reviewers to make a final decision about inclusion. During this full-text review stage, where necessary, study authors will be contacted for further information. At both the abstract and full-text screening stages, conflicts will be resolved by the two reviewers (BS and AH) first attempting to reach a consensus view; failing which, a third reviewer (NL) will be the final arbitrator. The study selection process will be summarised using a Preferred Reporting Items for Systematic Reviews and Meta-Analyses (PRISMA) flow diagram.

#### Sampling

We will use a combination of sampling techniques, including two types of ‘purposeful sampling’, a methodological approach that is particularly suitable for the focus of our scoping review [18]. These sampling techniques are intended to address both the breadth (for example, exploring the characteristics of studies on data harmonisation) and depth (for example, definitions, concepts, components, processes and explanations of casual mechanisms of data harmonisation) [18, 19] of the review.

For objective 1, we will not apply any sampling strategy, in order to ensure we capture the characteristics of the widest range of studies on the topic. For objectives 2 and 3, we will apply both maximum variation sampling (to identify both variation and similarities in definition and concepts and intervention descriptions) as well as theoretical sampling (where we will sample in relation to emerging theoretical insights and questions to provide a sufficiently ‘rich’ synthesis of descriptions of underlying causal mechanisms) [19]. The theoretical sampling will be iterative as we will start with synthesising emerging insights and may then loop back and look for more studies.

### Data extraction or ‘charting the data’

**Fig. 1:**
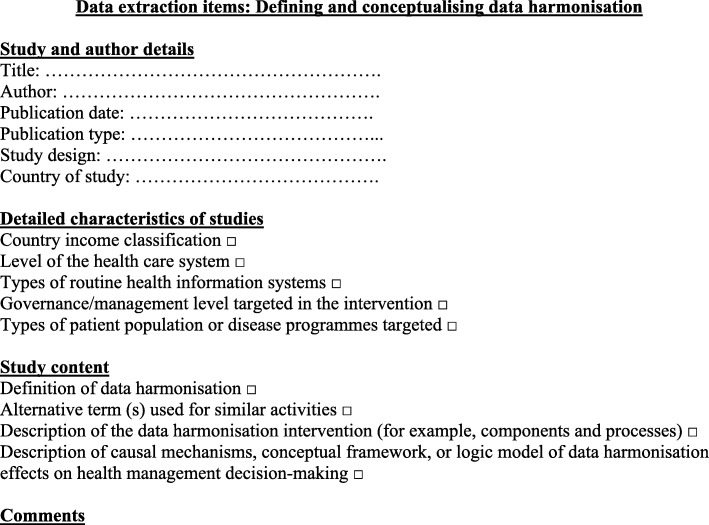
Data extraction framework

Once the list of papers to be included is finalised, the data extraction and sorting process (also referred to as ‘charting of data’ in Arksey and O’Malley) is the next step [15]. Data extraction of all the included studies will be conducted by one reviewer (BS), using the data extraction framework presented in Fig. 1. The extraction framework will be used to collect, sift and sort data that can address the three objectives of the review. This will be a mixture of general ‘demographic’ information about the study (such as country, level of the health care system) and specific information about the data harmonisation intervention (such as definitions, types of routine information systems, components, outcomes) and suggested causal mechanisms for the effect of data harmonisation on management decision-making. The framework will be piloted on the first few studies and revised where necessary. One other reviewer (AH) will independently conduct data extraction for a random sample of 10% of the included studies to increase reliability.

The process of data extraction and sorting will be done in Excel, using the data items in the data extraction framework (Fig. 1) to fill in information for each of the items in the framework. This will also allow for comparison of key items across studies and allow for synthesis within and across data items (for example, comparing definitions across studies, or comparing within one study, the definition and the description of the intervention components and processes).

As this scoping review aims to identify various characteristics, definitions and causal mechanisms of data harmonisation, we will not conduct any risk of bias or quality assessment of included studies. This approach is consistent with scoping reviews of similar aims and methodological frameworks for conducting scoping reviews [15, 20, 21].

#### Data synthesis or ‘collating, summarising and reporting the findings’

One review author (BS) will conduct data analysis, using manual coding and data synthesis methods on the extracted data from included studies. Another reviewer (NL) will review the data analysis work on an ongoing basis as an additional quality check.

This review will combine quantitative and qualitative syntheses to provide an overview of our findings. First, we will present an overview of all the included studies using a numerical analysis of the key characteristics of the studies [15]. The numerical synthesis will include following categories: income level of the country, the level of the health care system targeted in the intervention (for example primary health care, hospital-level, community-based health care), the particular type of routine health information systems involved (for example, clinical care, finance, human resources or drug supply information systems), the governance/management level targeted in the intervention (for example facility, district, regional or national levels) and types of patient population or disease programme (for example non-communicable disease or adult reproductive health).

The second synthesis approach will be a qualitative narrative synthesis [21] of data harmonisation definitions and of the conceptual models for understanding of how data harmonisation is meant to improve health management decision-making. We will collate and summarise definitions of data harmonisation and related concepts describing data harmonisation activities by looking for the key components across definitions and for key variations. We will code and synthesise the extracted data to identify the key issues that emerge regarding components and processes of data harmonisation interventions, the expected outcomes and impacts, and the factors influencing data harmonisation effects on management decision-making (including the steps of production, access and/or utilisation of health information).

To summarise, the numerical and narrative synthesis will result in three sets of findings: (a) an overview of key characteristics of data harmonisation studies, (b) the definitions and conceptualisations of data harmonisation, and (c) a narrative synthesis of the relationship between data harmonisation and health management decision-making.

Finally, we will ensure that the reporting of our findings is aligned with the PRISMA 2015 statement presented in Additional file 2.

#### Ethics and dissemination

This is a scoping review of completed studies, so no ethical approval is required. The results will be disseminated through peer-reviewed publications and conference presentations as well as shared with local and national stakeholders engaged in data harmonisation projects.

## Discussion

To our knowledge, this scoping review will be the first to synthesise definitions and conceptualisations of data harmonisation in the literature, as well as the underlying explanations in the literature of the causal links between data harmonisation and health management decision-making. Given time and financial constraints, we will only search for English studies published after 2000; potentially relevant studies may be missed. Applying purposeful sampling techniques will assist with addressing both breadth and depth of explanation in this scoping review, but it may also result in missing potentially useful content [18, 19]. This scoping review will be of interest to designers, implementers and users of data harmonisation interventions; it will broaden understandings of the range and complexity of studies, definitions, systems, organisations and stakeholders involved in such interventions and of the intended causal pathways for improving health management decision-making.

## Additional files

Additional file 1: Search strategy developed in PubMed database. (DOCX 14 kb)

Additional file 2: PRISMA-P 2015 Checklist. (DOCX 32 kb)

## Abbreviations

HIE: Health information exchange
LMICs: Low- and middle-income countries
MeSH: Medical Subject Headings
PRISMA: Preferred Reporting Items for Systematic Reviews and Meta-Analyses
RHIS: Routine health information system
WHO: World Health Organization
