# Supplementary material for: Defining and conceptualising data harmonisation: a scoping review protocol
Source: Syst Rev. 2018 Dec 6;7:226. doi: 10.1186/s13643-018-0890-7 (PMC6284298; doi:10.1186/s13643-018-0890-7)
Supplement: Supplementary file 1 — Search strategy developed in PubMed database. (DOCX 14 kb) [file 13643_2018_890_MOESM1_ESM.docx]

**Additional file 1.** Search strategy developed in PubMed database

| **Search strings** |
| --- |
| **Concept A: data harmonisation**  Search ((((((health information exchange) OR data harmonization) OR data integration) OR information exchange) OR information dissemination)) OR ((((((("Data Curation"[Mesh]) OR "Data Warehousing"[Mesh]) OR "Medical Informatics"[Mesh]) OR "Data Mining"[Mesh]) OR "Information Dissemination"[Mesh]) OR "Health Information Management"[Mesh]) OR "Health Information Exchange"[Mesh]) |
| **Concept B: health information systems (HISs)**  Search (((((((electronic medical record) OR health information management) OR hospital information management system) OR district health information system) OR district health management information system) OR healthcare information system)) OR (((("Electronic Health Records"[Mesh]) OR "Health Records, Personal"[Mesh]) OR "Health Information Systems"[Mesh]) OR "Medical Records Systems, Computerized"[Mesh]) |
| **Concept A + Concept B:**  Search ((((((((health information exchange) OR data harmonization) OR data integration) OR information exchange) OR information dissemination)) OR ((((((("Data Curation"[Mesh]) OR "Data Warehousing"[Mesh]) OR "Medical Informatics"[Mesh]) OR "Data Mining"[Mesh]) OR "Information Dissemination"[Mesh]) OR "Health Information Management"[Mesh]) OR "Health Information Exchange"[Mesh]))) AND ((((((((electronic medical record) OR health information management) OR hospital information management system) OR district health information system) OR district health management information system) OR healthcare information system)) OR (((("Electronic Health Records"[Mesh]) OR "Health Records, Personal"[Mesh]) OR "Health Information Systems"[Mesh]) OR "Medical Records Systems, Computerized"[Mesh])) |
| **Filters:**  Publication date and English language:  ("2000/01/01"[PDat] : "2018/07/31"[PDat]) AND Humans[Mesh] AND English[lang]))))) AND ((((public or public sector))) OR "Public Sector"[Mesh])  Study design:  Case Reports[ptyp] OR Clinical Study[ptyp] OR Clinical Trial[ptyp] OR Comparative Study[ptyp] OR Controlled Clinical Trial[ptyp] OR Evaluation Studies[ptyp] OR Historical Article[ptyp] OR Interview[ptyp] OR Multicenter Study[ptyp] OR Observational Study[ptyp] OR Practice Guideline[ptyp] OR Pragmatic Clinical Trial[ptyp] OR Randomized Controlled Trial[ptyp] OR Review[ptyp] OR systematic[sb] OR Technical Report[ptyp] |
